# The Effect of Oral Intake of Low-Temperature-Processed Whey Protein Concentrate on Colitis and Gene Expression Profiles in Mice

**DOI:** 10.3390/foods3020351

**Published:** 2014-06-13

**Authors:** Sharmila Jayatilake, Katsuhito Arai, Nanami Kumada, Yoshiko Ishida, Ichiro Tanaka, Satoru Iwatsuki, Takuji Ohwada, Masao Ohnishi, Yoshihiko Tokuji, Mikio Kinoshita

**Affiliations:** 1 United Graduate School of Agricultural Sciences, Iwate University, Ueda 3-18-8, Morioka, Iwate 020-8550, Japan; E-Mails: sharmilaeas@gmail.com (S.J.); s17046@st.obihiro.ac.jp (K.A.); taku@obihiro.ac.jp (T.O.); mohnishi@fujijoshi.ac.jp (M.O.); kinosita@obihiro.ac.jp (M.K.); 2 Department of Food Science, Obihiro University of Agriculture and Veterinary Medicine, Inada-cho W2-11, Obihiro, Hokkaido 080-8555 Japan; E-Mails: gekogeko773@yahoo.co.jp (N.K.); y-ishida@gh-assoc.ne.jp (Y.I.); tanaka-ichiro@outlook.jp (I.T.); 3 Asama Chemical Co. LTD., Nihonbashi-kodenma-cho 20-3, Chuo-ku, Tokyo 103-0001, Japan

**Keywords:** colitis, anti-inflammation, whey protein, DNA microarray, immunomodulation

## Abstract

Inflammatory bowel disease (IBD) is an autoimmune disease of unknown etiology and can lead to inflammation and cancer. Whey proteins contain many bioactive peptides with potential health benefits against IBD. We investigated the effect of low-temperature-processed whey protein concentrate (LWPC) on the suppression of IBD by using a dextran sodium sulfate (DSS)-induced colitis model in BALB/c mice. Oral intake of LWPC resulted in improved recovery of body weight in mice. Histological analysis showed that the epithelium cells of LWPC-treated mice were healthier and that lymphocyte infiltration was reduced. The increase in mucin due to the LWPC also reflected reduced inflammation in the colon. Transcriptome analysis of the colon by DNA microarrays revealed marked downregulation of genes related to immune responses in LWPC-fed mice. In particular, the expression of interferon gamma receptor 2 (Ifngr2) and guanylate-binding proteins (GBPs) was increased by DSS treatment and decreased in LWPC-fed mice. These findings suggest that LWPCs suppress DSS-induced inflammation in the colon by suppressing the signaling of these cytokines. Our findings suggest that LWPCs would be an effective food resource for suppressing IBD symptoms.

## 1. Introduction

Inflammatory bowel disease (IBD), including Crohn’s disease (CD) and ulcerative colitis (UC), is a chronic autoimmune disease condition, the pathogenesis of which is unknown [1]. IBD is considered to occur due to a combination of genetic, environmental, microbial and immunological factors characterized by the pronounced destruction of the gastrointestinal mucosa and the epithelial cell barrier. Subsequent infiltration of luminal antigens activate macrophages and T lymphocytes, which stimulate the production of proinflammatory cytokines, such as interleukin 1 (IL-1), interferons and tumor necrosis factor α (TNF-α), giving rise to a cascade of inflammatory reactions [2]. Manifestations of IBD include diarrhea, cramping, abdominal pain and fever [3]. Prolonged exposure to IBD is known to lead to colorectal cancer, which is one of the most common cancers, accounting for about 100,000 hospitalizations per year in the U.S. alone, and the incidence of IBD has continued to increase in recent decades in both developed and developing countries [4]. Current treatments for IBD typically include standard drug treatments, such as anti-inflammatory drugs, immune modifiers and antibiotics, and are frequently associated with severe side effects [5], which indicate the need for new and safe medicaments to contribute to the prevention and control of this disease.

Recent research reveals that food-derived peptides from various sources, including seafood, milk and plants, contain bioactive functional properties, especially in the suppression of colitis. Young *et al.* [6] showed that soy-derived peptides have the ability to suppress dextran sodium sulfate (DSS)-induced colitis through reducing the anti-inflammatory mediators. A novel purified peptide from pacific oyster (*Crassostrea gigas*) has been shown to reduce inflammation through immunomodulation [7]. Milk is known to be a rich source of nutrients coupled with bioactive properties that supports the growth and development of infants. Whey protein, a by-product of the cheese-making process, represents approximately 20% of the total protein content in bovine milk. The major protein components in whey are β-lactoglobulin, α-lactalbumin, immunoglobins, serum albumin, lactoferrin and lactoperoxidase [8]. Bioactive peptides are released from native protein during the course of gastric digestion. Most of such whey peptides have shown interactions directly with the immune cells, thereby modulating the immune system [9], or indirectly acting upon stimulating beneficial bacteria in the gut, cardiovascular health [10], improved muscle strength, inhibition of osteoporosis, anti-inflammatory, antibacterial and anticancer effects [11,12]. Both β-lactoglobulin and α-lactalbumin have been shown to inhibit colon carcinoma [13,14,15]. Whey peptides, such as β-casomorphins and α-lactorphins, derived from milk enhance the secretion of mucin from goblet cells via upregulation of the mucin gene [16], showing protective effects through maintaining gut homeostasis and suppressing intestinal inflammation. Recent work reveals a milk-derived glycomacropeptide, which shows anti-inflammatory functions in colitis mouse via modulation of macrophages and Th1 and Th17 lymphocytes. This modulatory effect was observed to arrest the signal transducers and activators of transcription (STAT) pathway and to suppress interferon gamma (IFNγ) expression [17]. These studies indicate the importance of whey proteins as an alternative medicament for the suppression of IBD. However, the mechanism of action or exact therapeutic use of whey proteins against IBD has not been clearly evaluated to date.

The functional properties of cheese whey protein are known to differ from the properties of raw milk based on the sterilization method applied during processing. The milk pasteurization process is known to cause a low degree of protein denaturation; thus, whey generated by pasteurized milk processing has been reported to have more bioactive functions when compared to whey processed at higher temperatures. Li *et al*. [18] showed that a minimally processed whey protein concentrate (heat treated, <40 °C) had positive effects on intestinal structure, function and integrity compared with whey proteins treated at higher temperatures. Elfstrand *et al.* [19] found the recovery of immunoglobins to be increased by low-temperature-treated colostrum compared to the findings for colostrum prepared by standard higher temperature treatments. 

Therefore, the present study was conducted to evaluate the effects of low-temperature-treated whey proteins in relation to their suppression of colon inflammation in the dextran sulfate sodium (DSS) mouse model of experimental colitis. The possible mechanisms by which whey protein may exert its action were studied via DNA microarrays followed by a comparison of the gene expression levels.

## 2. Experimental Section

### 2.1. Preparation of Whey Protein

The low-temperature-processed whey concentrate (LWPC) powder was a commercial product kindly gifted by Asama Chemical Co. Ltd. (Tokyo, Japan) The LWPC was dissolved in distilled water and heated at 70 °C for 2 h, and this solution was concentrated by freeze-drying. The resulting powder is named high-temperature-processed whey protein concentrate (HWPC). The protein profiles of HWPC and LWPC were analyzed by SDS-polyacrylamide gel electrophoresis [20] with 5%–20% gradient gels, followed by Coomassie brilliant blue staining.

### 2.2. Animals and Diets

The two treatment diets were prepared based on the AIN-76 diet (the composition is given in the Supplementary Materials) [21,22], where 50% of the casein in the AIN-76 diet was replaced separately with each of the above-described processed whey protein concentrates, HWPC or LWPC. Normal AIN-76 was used as a control diet.

Female BALB/c mice (4 weeks old) were obtained from CREA Japan Inc. (Tokyo, Japan) and housed in isolated cages at 20 °C under a 12 h light/dark cycle. After 10 days of acclimatization with the AIN-76 diet and water provided *ad libitum*, the mice were randomly divided into 4 experimental groups (5 mice per group). Colitis was induced in Groups 1 to 3 through the administration of 2.5% dextran sulfate sodium (DSS) in the drinking water [23], and each group was separately fed the AIN-76 basal diet, AIN-76 + HWPC or AIN-76 + LWPC in parallel with the induction of colitis. Group 4 was untreated. After a period of 14 days, DSS administration was arrested, and the experiment was continued until Day 21. On Day 21, the mice were sacrificed under ether anesthesia, and the portion of the large intestine from the cecum to the vent was removed and rinsed in cold saline. All protocols involving animals were approved by the Institutional Animal Care and Use Committee (20-101) and conducted according to the guidelines of Obihiro University of Agriculture and Veterinary Medicine.

### 2.3. Histopathological Experiments

Five colon tissue samples were obtained from each treatment group representing all 5 mice. Each tissue sample was taken from a different site so as to represent the whole column within a treatment group. The colon tissues were fixed overnight in 4% paraformaldehyde in phosphate-buffered saline, embedded in paraffin wax and sectioned (10 μm) using a microtome, followed by staining with hematoxylin and eosin (HE). Twenty-five slide spots from each treatment group were used for microscopical examination with 5 spots representing each tissue section. The slides were viewed under a light microscope. The slides were further evaluated with regard to histological damage to the colon using a semi-quantitative scoring system (Table 1) with minor alterations to the method used by McCafferty *et al.* [24], with a maximum possible total damage score of 18 when summed. Three semi-trained panelists performed the scoring under the guidance of a trained pathologist. Scores obtained for different treatments were analyzed using a non-parametric one-way ANOVA in STATA version 11.1 followed by Tukey HSD pairwise comparisons. 

**Table 1:** Parameters and scores used to assess damage in the colon tissues of mice.

Parameters	Scores			
	0	1	2	3
Extent of damage to colon structure	normal	mild	moderate	extensive
Crypt atrophy	not present	mild	moderate	extensive
Degree of cellular infiltration	normal	mild	moderate	extensive
Extent of muscle thickening	normal	mild	moderate	extensive
Extent of crypt abscess	not present	mild	moderate	extensive
Goblet cell depletion	normal	mild	moderate	extensive

The final score allocated is the average value from 25 slide spots from each different treatment.

### 2.4. Western Blotting

The mouse colon was homogenized, and proteins were extracted with general SDS-PAGE sample buffer. The protein concentration was assessed using the Bio-Rad protein assay method. Equal amounts of each extract were then subjected to protein separation on SDS-PAGE (15% gel) and then electro-transferred onto a nitrocellulose membrane. The membrane was blocked with Tris-buffered saline and 6% milk protein and then incubated overnight at 4 °C with primary rabbit antibodies for mucin 2 (Cosmobio, Tokyo, Japan), followed by incubation with a secondary antibody for rabbit IgG labeled with horse radish peroxidase. Detection was performed using the ECL system (GE Healthcare, Little Chalfont, UK). 

### 2.5. DNA Microarray and RT-QPCR

Colon tissues were ground in liquid nitrogen, followed by RNA extraction using the SV Total RNA isolation system (Promega, Madison, WI, USA) according to the manufacturer’s protocol. The extracted RNA was checked by spectrophotometer analysis and electrophoresis and then stored at −80 °C for further use.

RNA samples from each treatment replicate were pooled, and 500 ng of RNA from each treatment condition were used for transcriptome analysis of the mouse colon using a GeneChip 430A 2.0 mouse array (Affymetrix, Santa Clara, CA, USA). Experiments were performed according to the manufacturer’s technical manual (Affymetrix). Data obtained from scanning the arrays were normalized (robust multi-array analysis; RMA) and analyzed using Arraystar software (DNAStar, Madison, WI, USA). Genes that were up- or down-regulated by ≥2-fold in comparison with the DSS treatment were categorized using the Database for Annotation, Visualization and Integrated Database (DAVID) version 6.7 [25,26] and the annotation data of “biological processes” in Gene Ontology [27].

To validate the changes in the expression of the selected genes, we performed quantitative real time RT-PCR (QPCR). We selected guanylate binding protein 1 (Gbp1:NM_010259), Gbp2 (NM_010260) and interferon gamma receptor 2 (IFNgr2: NM_008338) for QPCR analysis. cDNA was synthesized using Superscript II reverse transcriptase (Life Technologies, Rockville, MD, USA) and oligo-dT primers. Primers for the genes were selected from PrimerBank [28,29] as described in Table 2. QPCR was performed with the specific primers and SYBR premix Ex Taq II (Takara, Kyoto, Japan) on an ABI 7300 Real-time PCR system (Applied Biosystems, Foster City, CA, USA), and the results were normalized using expression levels of the glyceraldehyde-3 phosphate dehydrogenase gene (GAPGH: NM_008084). All data are presented as the mean ± standard deviation (SD). Statistical analysis was performed by using the Student’s *t*-test.

**Table 2:** Primer sequences used for QPCR analysis.

Gene symbol	GenBank accession	Primer BankID	Primer sequence (5ʹ–3ʹ)	Amplicon size
			Forward primer/reverse primer	
Gbp1	NM_010259	6753948a1	ACAACTCAGCTAACTTTGTGGG TGATACACAGGCGAGGCATATTA	183
Gbp2	NM_010260	6753950a1	CTGCACTATGTGACGGAGCTA GAGTCCACACAAAGGTTGGAAA	115
IFNgr2	NM_008338	6680373a1	TCCTCGCCAGACTCGTTTTC GTCTTGGGTCATTGCTGGAAG	115
GAPDH	NM_008084	6679937a1	AGGTCGGTGTGAACGGATTTG GGGGTCGTTGATGGCAACA	123

## 3. Results and Discussion

### 3.1. Heat Treatment Affects the Characteristics of Whey Proteins

**Figure 1:**
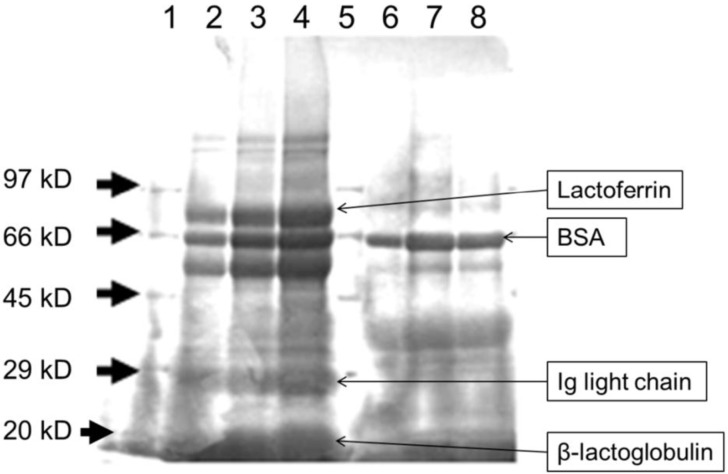
SDS-PAGE profiles of whey protein concentrates. Lanes 1 and 5: marker; lanes 2–4: low-temperature-processed whey protein concentrate (LWPC); lanes 6–8: high-temperature-processed whey protein concentrate (HWPC).

The protein profiles of LWPC and HWPC were compared by SDS-PAGE (Figure 1). LWPC showed clear peptide bands of high intensity at approximately 20, 29 and within 60–80 kD. These molecular weights represent β-lactoglobulin (20 kD), light-chain immunoglobins (29 kD), and bovine serum albumin (BSA) (66 kD) and lactoferrin (77 kD), as confirmed by Jimenez *et al.* [30] and Morin *et al.* [31]. In the HWPC lanes, proteins within the range of 60–80 kD were the most prominent, but presented a lower intensity than that in the LWPC lanes. The clearest band coincided with BSA. Both lactoferrin and light-chain immunoglobins have degraded in the HWPC lane. These proteins contain immunomodulatory, anti-inflammatory and antibacterial functions. Further, they show rapid degradation at temperatures above 65 °C. Similar results were shown in [30]. It is clear that whey protein degradation increased under the higher-temperature treatment. Jovanovic *et al.* [32] obtained the same finding, where a gradual decrease in all whey proteins was observed with treatments at increasing temperatures. Lin *et al.* [33] also obtained similar results for protein degradation with increasing temperature; however, BSA was stable at temperatures lower than 80 °C, which may explain the presence of BSA in HWPC treated at 70 °C in the present study. β-Lactoglobulin, BSA, lactoferrins and immunoglobins are known to have bioactive properties important for treating many diseases [34].

### 3.2. Oral Intake of LWPC Protects against DSS-Induced Colitis in Mice and Enhances Recovery from Colitis

**Figure 2:**
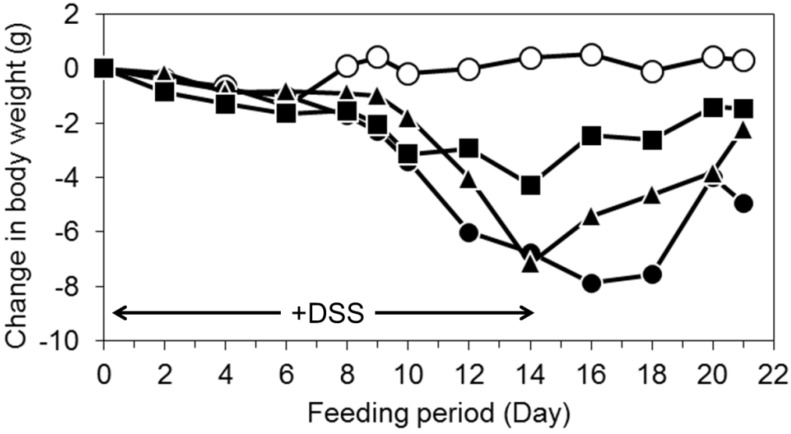
The effect of the oral intake of whey protein concentrates on body weight of dextran sodium sulfate (DSS)-treated mice. Opened circles, normal mice without DSS treatment; filled circles, DSS-treated mice; filled triangles, mice treated with DSS + HWPC feed; filled squares, mice treated with DSS + LWPC feed.

DSS-induced intestinal injury serves as an experimental model for human ulcerative colitis and is a well-established method for the chemical induction of intestinal injury [35]. The change in body weight in the mice during the experimental period is shown in Figure 2D. The body weight of mice without DSS treatment was constant throughout the experiment period. DSS induction started to show effects by Day 8 after treatment in DSS-treated mice; these mice continued to lose weight even after DSS administration was stopped (Days 14–16). Reduction of body weight is a common effect seen in DSS colitis and is caused by reduced food intake and impaired intestinal nutrient absorption [36]. The body weight of DSS + LWPC- and DSS + HWPC-treated mice showed a reduction until Day 14. DSS + LWPC-treated mice lost less weight than DSS- and DSS + HWPC-treated mice. After the DSS treatment was stopped, the body weight of the mice steadily increased. This recovery of weight was most prominent in LWPC-treated mice. These findings indicate that LWPC treatment resulted in amelioration of DSS-induced colitis and facilitated rapid recovery from colitis after the administration of DSS was stopped. The role of LWPC in suppressing DSS-induced colitis has not been clearly determined; however, Sprong *et al*. [37] found that DSS-induced colitis in mice was ameliorated by feeding the mice cheese whey proteins, which stimulated gastric mucin expression and intestinal microflora modulation.

### 3.3. LWPC Protects the Intestinal Epithelium against DSS-Induced Inflammation

**Figure 3:**
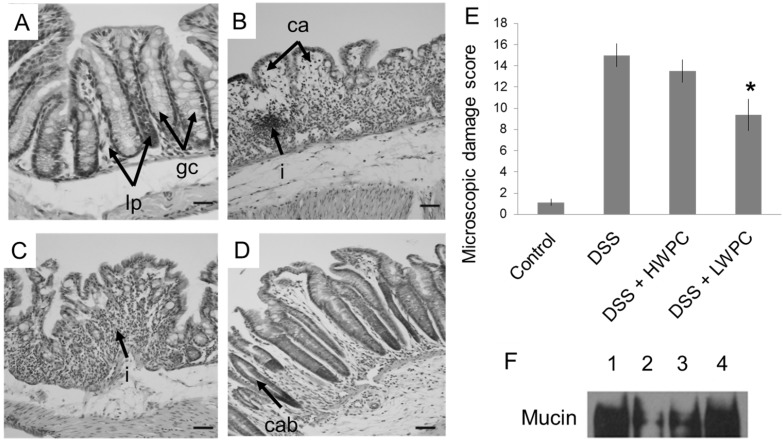
Histological effects of whey protein intake on DSS-induced colitis. (**A**–**D**) Transverse colon sections of mice on Day 21, stained with HE. (**A**) Normal mice without DSS treatment; (**B**) DSS-treated mice; (**C**) DSS mice treated with DSS + HWPC feed; (**D**) mice treated with DSS + LWPC feed (lp, lamina propria; gc, goblet cells; i, infiltration of leukocytes; ca, crypt atrophy; cab, crypt abscess; bars = 10 μm); (**E**) histological damage scores for the mouse colon on Day 21, evaluated according to Table 1. The final scores allocated are the average values of 25 slide spots of each different treatment group. * Statistically significant difference from the DSS group (*p* < 0.05). (**F**) Expression analysis of myeloperoxidase (MPO) and mucin in colon by western blot. Lane 1: normal mice without DSS treatment; lane 2: DSS-treated mice; lane 3: mice treated with DSS + HWPC feed; lane 4: mice treated with DSS + LWPC feed.

Under inflammatory conditions, excess infiltration of neutrophils can be observed clearly. Microscopic images of colon sections from mice subjected to different treatments are shown in Figure 3. The normal mucosal structure of the colon (Figure 3A) demonstrated the clear separation of crypts and the lamina propria, and infiltration of leucocytes was not observed. Epithelial cells and goblet cells were prominent within the lining of each crypt. Histopathological features of inflammation include abnormal crypt formation, goblet cell depletion and marked intestinal wall thickening, with leukocyte infiltration [24]. DSS treatment induced severe inflammation of the colon with complete loss of the mucosal structure, severe infiltration of the lamina propria and thickening of the intestinal walls (Figure 3B). The colon of DSS + HWPC-treated mice showed a slight improvement in the structure of the mucosa; however, only a few goblet cells remained in crypts with an abnormal size and shape, and severe leukocyte infiltration was present (Figure 3C). DSS + LWPC treatment (Figure 3D) produced a distinct reduction in the severity of inflammation compared to that observed for DSS + HWPC and also improved the mucosal structure. The crypt structure and shape were well maintained, although the number of goblet cells decreased, and leukocyte infiltration was moderate. To evaluate the extent of damage in the colon sections, we scored the sections based on visual criteria (Table 1). The score of the colon tissue in DSS-treated mice was 15.0, whereas the score in colon tissue from mice without DSS treatment was 1.1 (Figure 3E). DSS + LWPC-treated mice (9.4) showed a significant decrease in histological damage to the colon compared to that in DSS-treated mice. However, a significant reduction of damage scores was not observed in the DSS + HWPC group (13.3). These results suggest that oral intake of LWPC is effective for protection from DSS-induced colitis or for regeneration of damaged colon tissue. Moreover, pasteurization at a higher temperature may cause whey protein to lose these favorable properties. 

In the colon, mucins are produced by goblet cells for lubrication [38]. The availability of mucins in the colon epithelium is an indicator showing that goblet cells are healthy and functioning well. Mucin was highly detected in mice colons without DSS treatment, and mucin levels were reduced by DSS treatment, indicating that goblet cells were destroyed by DSS-induced colitis in the colon (Figure 3F). Oral intake of both HWPC and LWPC increased the mucin level in the presence of DSS. Compared to the findings for the HWPC group, the expression level of myeloperoxidase (MPO) for the LWPC treatment was lower and was similar to that in the mice without DSS treatment (data not shown). The reduced MPO levels in the LWPC-treated mouse colons compared to the levels in the colons of control and HWPC-treated mice show that LWPC treatment reduces neutrophil recruitment into the colon, further suggesting that inflammation was suppressed. Similarly, the increase of mucin levels in the gastric epithelium indicates the recovery of the DSS-induced colon by regenerated epithelial cells and goblet cells. Compared with LWPC, HWPC had lower effects on reducing MPO activity and increasing mucin content in the colon. Sprong *et al*. [37] reported that cheese whey protein feed increased the mRNA levels of mucin 2 (MUC2) in the colon of rats induced with DSS inflammation, possibly because whey protein contains higher amounts of amino acids required for mucin synthesis. The quantity and composition of amino acids are almost identical between LWPC and HWPC. However, in our study, the protein profile was different according to the results of SDS-PAGE (Figure 1). LWPC is shown to retain more proteins than the HWPC fractions. These proteins possibly contain more bioactive peptides in a higher solubility form, which is more accessible in digestion. Bounous *et al.* [39] and Hongsprabhas *et al.* [40] also found similar results where mild heat treatment on whey proteins preserved the biological activity and showed an increased function of antioxidant and immune response, both *in vivo* and *in vitro*.

### 3.4. The Effect of LWPC on Gene Expression under DSS-Induced Inflammation in the Colon

**Figure 4:**
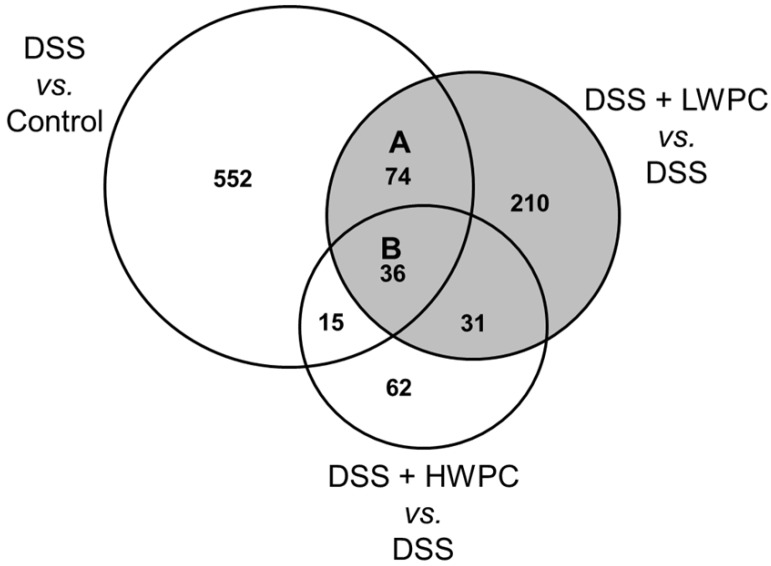
Venn diagram of differentially-regulated genes. Numbers show genes up- or down-regulated (≥2-fold), compared between the control (no treatment) and DSS, between DSS and DSS + LWPC and between DSS and DSS + HWPC groups.

To investigate the mechanism underlying the effect of LWPC, we evaluated the transcriptome of the mouse colon by DNA microarray analysis. Compared with the control group, 677 out of 22,626 probed genes were up- or down-regulated by over two-fold in the DSS treatment group (Figure 4, Table 3). Compared with the DSS group, the number of genes differentially expressed (≥2-fold change) in the LWPC group was higher (351) than that in the HWPC group (144). This finding suggests that LWPC has more bioactive peptides than HWPC. In total, 110 genes were regulated by both DSS and LWPC, whereas 51 genes were regulated by both DSS and HWPC. Therefore, LWPC may have a higher effect on the expression of genes involved in DSS-induced inflammation.

**Table 3:** The number of genes differentially regulated by DSS and whey protein concentrates.

Treatment group	Number of genes differentially regulated (fold change *vs*. control >2)		
	Upregulated	Downregulated	Total
DSS	348	328	677
DSS + LWPC	213	138	351
DSS + HWPC	113	31	144

**Figure 5:**
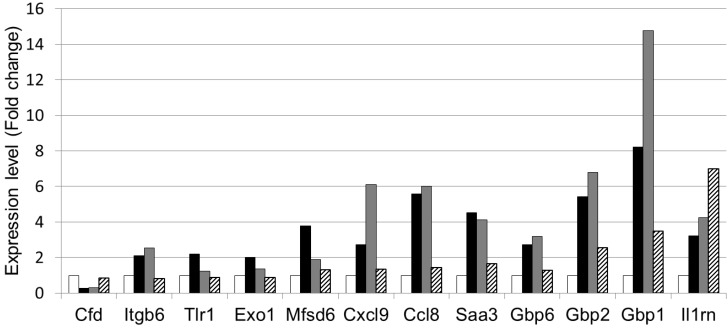
Expression of genes involved in the immune response that were differentially regulated by whey protein concentrate. The graph shows the relative expression level (fold change) determined by microarray analysis (white: normal mice without DSS treatment; black: DSS-treated mice; grey: mice treated with DSS + HWPC feed; hatched: mice treated with DSS + LWPC feed). The proper names of the genes are shown in Table 5.

**Figure 6:**
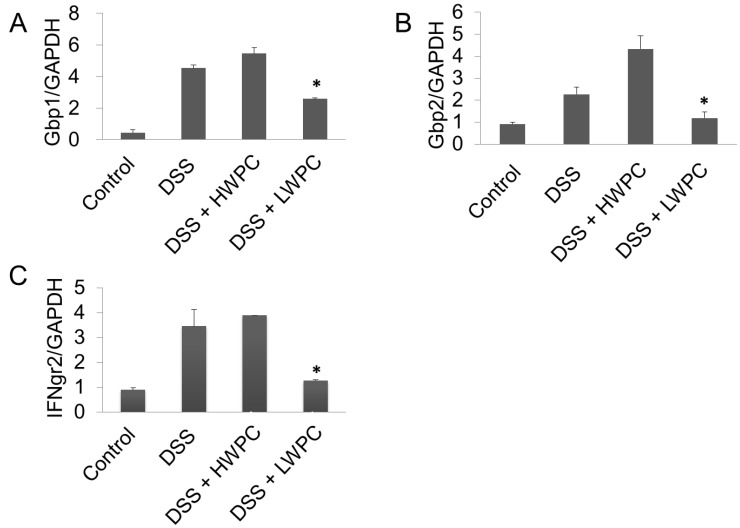
Gene expression analysis by real-time QPCR. The relative expression levels of Gbp1, Gbp2 and IFNgr2 are shown as ratios compared with GAPDH. * Statistically significant difference from the DSS group (*p* < 0.05).

The 110 genes regulated by both DSS and LWPC were further classified into functional groups using the DAVID functional annotation program according to the GO biological process in the GO ontology, as shown in Table 4. Among the genes, 12 genes were involved in “immune response” and sox genes were involved in “inflammatory response”, indicating possible effects on inflammation. The expression levels of the 12 immune responsive genes (Table 5) are shown in Figure 5. All of the genes were upregulated by DSS treatment and downregulated by LWPC treatment, except for Cfd (complement factor D) and Il1rn (interleukin 1 receptor antagonist). The genes encoding guanylate-binding proteins (GBP), such as Gbp1, Gbp2 and Gbp6, were highly expressed in the colon of DSS-treated mice, but distinctly downregulated by LWPC ingestion, whereas the expression levels did not decrease upon oral administration of HWPC. Our results revealed relative expression increases of 3.5-, 2.5- and 1.7-fold in LWPC-treated mice and 8.2-, 5.4- and 3.3-fold in DSS-treated mice for Gbp1, Gbp2 and Gbp6, respectively, relative to the blank treatment (Figure 6), which clearly indicates the reduced expression of Gbps in LWPC. Furthermore, Cxcl9 (chemokine (c-x-c motif) ligand 9), Ccl8 (chemokine (c-cl motif) ligand 8) and Itgb6 (integrin beta 6) showed a pattern similar to that of Gbps. The remaining genes were downregulated in both the HWPC and LWPC groups. However, the level of decrease of the expression of these genes was lower in the HWPC group than in the LWPC group.

**Table 4:** Functional categories of genes differentially regulated by both DSS and LWPC

GO biological process	Number of genes (fold change > 2)	% *	*p*-Value **
GO:0006955 Immune response	12	12.2	3.1 × 10^−4^
GO:0006869 Lipid transport	5	5.1	4.6 × 10^−3^
GO:0010876 Lipid localization	5	5.1	6.0 × 10^−3^
GO:0015918 Sterol transport	3	3.1	7.4 × 10^−3^
GO:0030301 Cholesterol transport	3	3.1	7.4 × 10^−3^
GO:0006954 Inflammatory response	6	6.1	9.0 × 10^−3^
GO:0048593 Camera-type eye morphogenesis	3	3.1	3.0 × 10^−2^
GO:0042159 Lipoprotein catabolic process	2	2.0	3.4 × 10^−2^
GO:0034754 Cellular hormone metabolic process	3	3.1	3.5 × 10^−2^
GO:0010817 Regulation of hormone levels	4	4.1	3.6 × 10^−2^

* Differentially regulated genes/total genes in category; ** modified Fisher exact *p*-value (Expression Analysis Systematic Explorer score) adopted in the DAVID annotation chart.

**Table 5:** The genes in “immune response” (GO:0006955) whose expression was regulated by both DSS and LWPC.

GenBank Accession No.	Gene symbol	Gene description	Regulation byDSS and LWPC	
NM_013459	Cfd	Complement factor D (adipsin)	↓	↑
NM_001159564	Itgb6	Integrin beta 6	↑	↓
NM_001276445	Tlr1	Toll like receptor 1	↑	↓
NM_012012	Exo1	Exonuclease 1	↑	↓
NM_133829	Mfsd6	Major facilitator S domain containing 6	↑	↓
NM_008599	Cxcl9	Chemokine (c-x-c motif) ligand 9	↑	↓
NM_021443	Ccl8	Chemokine (c-cl motif) ligand 8	↑	↓
NM_011315	Saa3	Serum amyloid A3	↑	↓
NM_010259	Gbp1	Guanylate binding protein 1	↑	↓
NM_010260	Gbp2	Guanylate binding protein 2	↑	↓
NM_145545	Gbp6	Guanylate binding protein 6	↑	↓
NM_001039701	Il1rn	Interleukin 1 receptor antagonist	↑	↑

Quantitative real-time RT-PCR was performed to confirm the expression levels of Gbp1 and Gbp2 (Figure 6A,B). Both Gbp1 and Gbp2 were upregulated by DSS treatment by 4.54 ± 0.19- and 2.27 ± 0.32-fold, respectively. Oral intake of LWPC significantly decreased the expression level of both Gbp1 and Gbp2 in the colon of DSS-treated mice, with relative expression levels of 1.26 ± 0.35- and 1.18 ± 0.59- fold, respectively, relative to the blank treatment. However, HWPC did not suppress the expression of these Gbp genes. The GBP family is a group of proteins that bind to guanine nucleotides, but that are different from the G protein family. GBPs are highly expressed in endothelial cells and are strongly induced by inflammatory cytokines, such as interferon (IFN) gamma, tumor necrosis factor alpha, interleukin 1 (IL-1) alpha and IL-1beta [41]. Gbp1 also contributes to cell survival through the inhibition of apoptosis, thereby maintaining the integrity of the barrier function of the epithelium [42]. Gbp1 has also been proposed to regulate vital homeostatic functions, such as apoptosis and cell growth [43]. Britzen-Laurent *et al.* [44] found that Gbp1 mediates the anti-tumorigenic effects of IFN gamma, resulting in the inhibition of tumor growth in colorectal cancer (CRC). Thus, higher expressions of GBPs are expected in inflammatory CRC sites. Gbp1 and Gbp2 expression may be reduced upon LWPC treatment through the suppression of the IFN gamma-mediated inflammatory response. Our microarray findings also showed the expression of the chemokine, Cxcl9, which was also reported to be induced via IFN gamma signaling [45]. Thus, the decreased expression of Gbp1, Gbp2, Gbp6 and Cxcl9 may result from the suppression of IFN gamma signaling through oral intake of LWPC. IFN gamma is a critical mediator of the immune system and inflammatory responses mediated by activated T-cells. IFN gamma acts to enhance inflammation by recruiting the infiltration of leukocytes. Excess IFN gamma results in inflammatory disorders and is well established to regulate cell proliferation and apoptosis through the disassembly of tight junctions and the reduction of the rate of cell migration in the epithelium in IBD [46]. According to our microarray results, the expression level of IFN gamma was not changed by LWPC treatment. However, we noticed that the receptor for IFN gamma, namely IFNgr2, was downregulated in LWPC by 1.7-fold in our microarray results. The gene expression level of IFNgr2 was validated by QPCR, as shown in Figure 6C. In particular, IFNgr2 was upregulated by DSS treatment and downregulated by oral intake of LWPC, but not by that of HWPC. We also found that further downstream of the IFNG pathway, the signal transducers and activators of transcription1 (STAT1) was downregulated by 2.1 by LWPC, while HWPC showed no changes. Interleukin 10 (IL-10) is a unique cytokine having anti-inflammatory properties [47]. According to recent research findings [48], both Il-10 and interleukin 10 receptor 1 (IL-10R1) increased in response to higher levels of IFNg in tissues. Interleukin 27 (IL-27) is characterized as a cytokine with intestinal barrier protection through transcription of antibacterial and anti-inflammatory genes [49]. LWPC did not show any change on the IL-10, IL-27 or their receptor expression levels in our results. Therefore, we suggest that suppression of inflammation by LWPC was not induced through the anti-inflammatory cytokines above. Our histological study described above confirmed the reduced inflammation and damage in the DSS-induced colon when treated with LWPC. This result is in line with the reduced expression of IFNgr2, GBPs and Cxcl9. Therefore, we suggest that LWPC acts by downregulating IFN gamma receptor, resulting in decreased inflammation in DSS-induced colitis.

Whey protein isolates subjected to high hydrostatic pressure have been shown to affect the suppression of tumor necrosis factor-alpha, interleukin 8 and interleukin 18 in Caco-2 colon cancer cells [50]. Rusu *et al.* [51] showed that whey protein increases innate immunity by priming neutrophils. Attaallah *et al.* [52] reported that oral intake of whey protein hydrolysate protects rats against azoxymethane- and DSS-induced colon cancer. These studies suggest that whey protein modulates the immune system. In this study, we showed that LWPC suppresses DSS-induced colitis by suppressing the IFN gamma pathway. This anti-inflammatory activity was lost upon heat treatment of the whey protein, which suggests that the main active peptide of whey protein is heat labile. Some proteins in whey and peptides derived from these proteins have been reported to have bioactive properties, as reviewed by Smithers [53]. Lactoferrin, lactoperoxidase (LPO) and immunoglobulins have antimicrobial activity. Shin *et al*. [54] reported that DSS-induced inflammation was suppressed by oral administration of LPO in mice and also showed that LPO reduces IFN gamma expression. However, LPO may not be a candidate active peptide for anti-inflammation in LWPC, because LWPC did not downregulate IFN gamma expression, but rather reduced Infgr2 expression in our study. Transforming growth factor beta (TGF-beta) is a cytokine that controls proliferation, cellular differentiation and other functions in most cells. TGF-beta is also involved in the effects of whey protein and has been shown to ameliorate IFN gamma-induced intestinal barrier disturbance by upregulating claudin-4 (Cldn4) in human HT-29/B6 cells [55]. However, LWPC did not affect the expression level of Cldn4 in our microarray experiment (data not shown). Thus, we expect that the anti-inflammatory peptide in LWPC is not TGF-beta or LPO. The anti-inflammatory effect of LWPC was lost by heat treatment. However, there are no reports regarding the heat stability of these bioactive peptides in whey to date. Exploration of the active peptides is now ongoing.

## 4. Conclusions

DSS-induced colitis was prevented by oral intake of LWPC, which also enhanced the recovery from colitis. Comprehensive analysis of gene expression in the colon by DNA microarray analysis showed that GBPs were downregulated by LWPC intake. Expression of GBPs is known to be regulated by interferon gamma. IFNgr2, a receptor for interferon gamma, was also downregulated by LWPC, which suggests that oral intake of LWPC results in the suppression of IFN gamma-mediated pathways and leads to the suppression of inflammation.

## Supplementary Materials

Supplementary File 1
