# Supplementary material for: The Effect of Oral Intake of Low-Temperature-Processed Whey Protein Concentrate on Colitis and Gene Expression Profiles in Mice
Source: Foods. 2014 Jun 13;3(2):351–68. doi: 10.3390/foods3020351 (PMC5302365; doi:10.3390/foods3020351)
Supplement: Supplementary File 1 [file foods-03-00351-s001.docx]

**Supplementary Information**

**Table S1.** Composition of the experimental diets.

| **Composition** | **AIN-76 * (10%)** | **LWPC diet (%)** | **HWPC diet (%)** |
| --- | --- | --- | --- |
| Milk casein  LWPC  HWPC | 20.0  0  0 | 10.0  10.0  0 | 10.0  0  10.0 |
| DI-methionine | 0.3 | 0.3 | 0.3 |
| Corn-starch | 15.0 | 15.0 | 15.0 |
| Caster sugar | 50.0 | 50.0 | 50.0 |
| Cellulose powder | 5.0 | 5.0 | 5.0 |
| Corn oil | 5.0 | 5.0 | 5.0 |
| Vitamin mix | 1.0 | 1.0 | 1.0 |
| Mineral mix | 3.5 | 3.5 | 3.5 |
| Choline-bi-tartrate | 0.2 | 0.2 | 0.2 |

* See references [1,2].

**References**

1. Lien, E.L.; Boyle, F.G.; Wrenn, J.M.; Perry, R.W.; Thompson, C.A.; Borzelleca, J.F. Comparison of AIN-76A and AIN-93G diets: A 13-week study in rats. *Food Chem. Toxicol.* **2001**, *30*, 385–392.
2. Reeves, P.G.; Nielsen, F.H.; Fahey, G.C. AIN-93 purified diets for laboratory rodents: Final report of the American Institute of Nutrition Ad Hoc Writing Committee on the reformulation of the AIN-76A rodent diet. *J. Nutr.* **1993**, *123*, 1939–1951.

© 2014 by the authors; licensee MDPI, Basel, Switzerland. This article is an open access article distributed under the terms and conditions of the Creative Commons Attribution license (http://creativecommons.org/licenses/by/3.0/).
